# Supplementary material for: Causal interplay between lactose intolerance and gut microbiota: a combined bidirectional Mendelian randomization and in vivo validation study
Source: Front Nutr. 2026 Jun 1;13:1803337. doi: 10.3389/fnut.2026.1803337 (PMC13265576; doi:10.3389/fnut.2026.1803337)
Supplement: Supplementary file 1 [file Data_Sheet_1.zip › supplementary materials/Forward/leave one out cross validation/ebi-a-GCST90027572.finngen_R12_E4_LACTONAS.pdf]

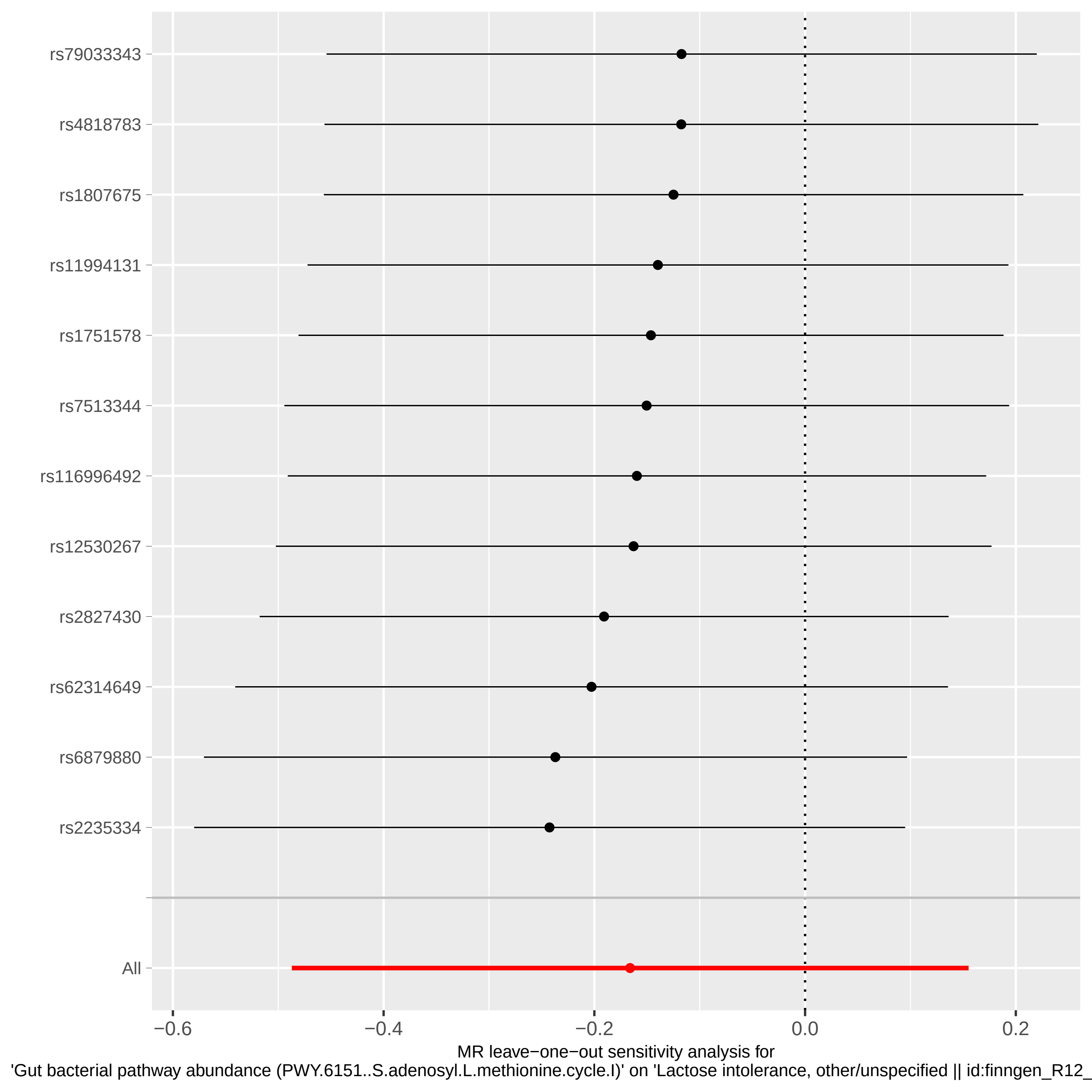

rs79033343
rs4818783
rs1807675
rs11994131
rs1751578
rs7513344
rs116996492
rs12530267
rs2827430
rs62314649
rs6879880
rs2235334
All
−0.6
−0.4
−0.2
0.0
0.2
MR leave−one−out sensitivity analysis for
'Gut bacterial pathway abundance (PWY.6151..S.adenosyl.L.methionine.cycle.I)' on 'Lactose intolerance, other/unspecified || id:finngen_R12_
